# Starch Hydrogels for Slow and Controlled-Release Fertilizers: A Review

**DOI:** 10.3390/polym17081117

**Published:** 2025-04-20

**Authors:** Andrés Felipe Chamorro, Manuel Palencia, Enrique Miguel Combatt

**Affiliations:** 1 Research Group of Electrochemistry and Environment (GIEMA), Faculty of Basic Sciences, Universidad Santiago de Cali, Cali 760035, Colombia; 2 Research Group in Science with Technological Applications (GICAT), Department of Chemistry, Faculty of Natural and Exact Science, Universidad del Valle, Cali 760032, Colombia; 3 Department of Agricultural and Rural Development, Faculty of Agricultural Sciences, Universidad de Córdoba, Monteria 230002, Colombia; ecombatt@fca.edu.co

**Keywords:** starch, hydrogel, fertilizer, agriculture, slow-release fertilizer

## Abstract

Fertilizers are widely used to increase agricultural productivity and ensure food security. However, their excessive use negatively impacts the environment, as a large portion is lost through leaching, degradation, and evaporation. Starch-based hydrogels (SHs) offer a promising alternative to mitigate these environmental effects by enabling the controlled release of nutrients. SHs are biodegradable, non-toxic, and biocompatible, making them attractive for agricultural applications such as soil remediation and fertilizer delivery. These materials consist of crosslinked, three-dimensional networks with high water absorption capacity. Their effectiveness in nutrient delivery depends on the synthesis method, nutrient source, and environmental conditions. While the literature on SHs is growing, most studies focus on laboratory-scale production, which limits their broader application in agriculture. This review aims to consolidate current knowledge on SHs and identify research gaps to guide the development of more efficient and environmentally friendly SH-based fertilizers. It provides an overview of SH formation methods, including graft copolymerization, chemical crosslinking, and physical interactions. Additionally, the review highlights SH applications in controlled fertilizer release, discussing encapsulation capacity, large-scale production techniques, and nutrient delivery in aqueous media, soils, seeds, and plants.

## 1. Introduction

Fertilizers play a crucial role in agriculture by promoting efficient crop growth. Their use has become increasingly necessary to meet the rising global food demand, which has grown by approximately three billion people over the past 40 years. The world population is projected to reach 9.7 billion by 2050 [1]. However, the continuous use of fertilizers has resulted in environmental degradation and economic losses, primarily due to high nutrient losses (40–70%), which contribute to serious ecological problems and hinder plant and crop development [2,3]. Controlled-release hydrogels and composites have emerged as promising alternatives to mitigate these limitations, primarily due to the presence of hydrophilic groups that can be readily modified to introduce new functionalities. These chemical groups also facilitate chemical and physical crosslinking reactions, forming a three-dimensional structure capable of absorbing and retaining large amounts of water. This crosslinked structure is a key feature, enabling a high nutrient retention capacity [4]. Most hydrogels and composites used in agriculture are derived from synthetic polymers such as polyacrylamides. However, these materials are not biodegradable and can negatively affect soil properties. To address these issues, biopolymers such as cellulose, chitosan (CS), alginate, collagen, and starch have been explored as sustainable alternatives. These biopolymers are biocompatible, biodegradable, and non-toxic, making them highly suitable for agricultural applications.

**Figure 1:**
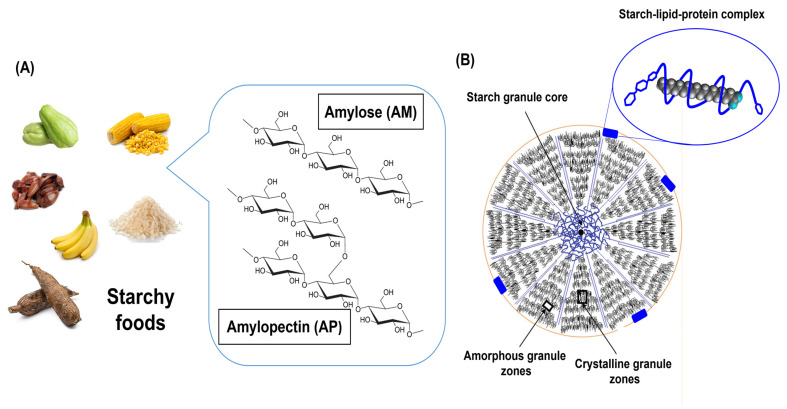
(**A**) Common starchy foods and the structures of AM and AP. (**B**) Model illustrating the distribution of amorphous and crystalline regions within a starch granule. Starch–lipid–protein complex is shown inside, which affects granule swelling. Modified from (**A**) [8]; (**B**) [9].

Hydrogels are attractive materials for nutrient delivery due to their high water absorption capacity, excellent stability after swelling and during storage, non-toxicity, and high gel fraction after crosslinking. These characteristics are essential for achieving controlled fertilizer release. Furthermore, hydrogels present a more cost-effective alternative compared to nanomaterials commonly used in agricultural applications [5]. SHs serve as both soil conditioners and yield enhancers by retaining water and fertilizers and gradually releasing them throughout crop development [5,6]. Among the biopolymers previously mentioned, starch (C_6_H_10_O_5_)_n_ stands out as one of the most abundant polysaccharides in nature, alongside cellulose and chitin. It is derived from various food sources such as cassava, rice, banana, and corn. Structurally, starch is a copolymer composed of amylose (AM) and amylopectin (AP) (Figure 1A), typically consisting of approximately 20–30% AM and 70–80% AP [7].

AM is a lightly branched polymer composed of a limited number of long glucan chains (ranging from 5 to 20), which tend to form helical structures. In contrast, AP is highly branched, consisting of numerous clusters of short chains [10]. The molecular weight (M_w_) of AP typically ranges from 10^7^ to 10^8^ g/mol [11]. However, the high M_w_ values reported in the literature are often attributed to aggregate formation in aqueous solutions. When dissolved in a good solvent such as dimethyl sulfoxide (DMSO), AP displays a lower M_w_, typically around 10^6^ g/mol [12]. These D-glucose polymers are organized into a complex hierarchical structure that gives rise to starch granules, which generally consist of alternating semicrystalline and amorphous growth rings (Figure 1B). The size of starch granules varies between 2 and 150 μm, depending on the botanical source. For instance, cereals tend to produce smaller granules compared to starches derived from roots and tubers [13].

In soils, SHs and their composites have been investigated for various applications, including (i) nutrient delivery, (ii) soil remediation, and (iii) protective coatings for herbicides and pesticides. Additionally, these materials contribute to improving several soil properties, such as enhancing permeability and water-use efficiency, extending irrigation intervals, increasing water retention across different soil types, minimizing soil erosion and runoff, reducing soil compaction, and improving drainage [14,15,16,17]. SHs have shown significant potential in the controlled transport of fertilizers, enabling their slow and sustained release in both plants and soils [18]. These hydrogels facilitate nutrient delivery by minimizing nutrient washout, reducing fertilization frequency, and limiting leaching through decreased runoff. Although numerous studies have explored the formation and application of SHs, many reviews have focused primarily on laboratory-scale production, often overlooking methodologies for large-scale manufacturing. Therefore, this review aims to provide a comprehensive overview of the characteristics and structure of SHs and their composites with potential agricultural applications. In particular, it emphasizes large-scale production strategies and summarizes controlled delivery systems for macronutrients such as nitrogen (N), phosphorus (P), and potassium (K) in soils and plants. These nutrients are applied in large quantities in crop cultivation, often resulting in substantial environmental and economic losses. The controlled delivery of these essential nutrients using SHs and their composites can help mitigate these issues by reducing runoff and leaching, enhancing fertilizer efficiency, and ultimately improving crop yields while minimizing environmental impact. The following section highlights various starch sources that can be used to develop cost-effective SHs for application in controlled fertilizer release.

## 2. Starch Sources and Chemical Composition

Several starch sources have been reported in the literature, primarily derived from fruits, stems, cereals, tubers, and roots. Starch is commonly isolated using the wet milling method, which involves soaking procedures at varying temperatures and the use of chemical agents such as alkalis or acids [13]. For example, in the extraction of starch from beans, a sodium sulfite solution is used during the soaking and blending stages. This facilitates water penetration into the starch granules while also preventing microbial growth. The resulting mixture is then filtered, and the suspension is decanted. This process is repeated, followed by resuspension in distilled water and centrifugation to obtain purified starch [19]. Similar methods are used to extract starch from quinoa [20], amaranth [21], and peas [22], typically involving an NaOH solution ranging from 0.1% to 0.3%. This alkaline treatment reduces the protein content through repeated filtration over approximately 15 days. In the case of lentils, starch extraction also involves an alkaline treatment; however, prior to this step, lentil flour is treated with an aqueous solution at pH 8 to solubilize proteins and facilitate starch isolation [23]. A different methodological modification was reported for extracting starch from sweet potato and cassava [13], where an NaHSO_3_ solution is used to inhibit the oxidation of metabolites such as tyrosine and dihydroxyphenylalanine. This prevents the formation of melanin, which imparts a red–brown coloration [13].

Table 1 presents various starch sources and their chemical composition, including the percentages of starch, protein, ash, lipids, and fibers. Many sources have high starch concentrations (>90%), such as cassava, banana, taro, yam, chestnut, unripe fruits, and loquat seeds. Thus, these starch sources, particularly cassava and banana, are optimal for producing SHs on a large scale, as they show high starch content and are produced in large quantities around the world. Conversely, some sources, such as corn and mango seeds, have been reported to contain low starch concentrations (<25%). According to the data, the AM concentration is not directly correlated with the total starch concentration. For example, longan contains 85.7% starch but has a higher AM concentration (26.4%) than cassava (16.71%). This suggests that the AM concentration varies depending on the source, ranging from 1.25% to 45.69%.

The concentrations of AM and AP influence the crystallinity of starch granules. These granules consist of a semi-crystalline structure composed of crystalline lamellae (closely packed glucan chains) and amorphous lamellae (non-ordered branching glucan chains) [24]. The organization of AM and AP forms rings or shells known as growth rings, which are constructed by alternating amorphous and semi-crystalline regions. These structures can be observed using a light microscope or a scanning electron microscope after treating the sample with a dilute acid solution [11]. Granule sizes are classified into three types based on their dimensions: A-type (>15 μm), B-type (5–15 μm), and C-type (<5 μm) [25]. Typically, all starch sources exhibit a wide granule size distribution within the same sample, containing different proportions of starch granule types. However, exceptions exist, such as cassava, potato, yam, loquat seed, and unripe fruits, where the granule size is predominantly classified as type A. This type is typically associated with a high AM content and low crystallinity [26,27]. The AM content increases as seeds mature and starch granules grow in size. For example, in corn endosperms, the AM content rises from 9.2% at 12 days after pollination to 24.2% at 30 days after pollination [28].

Starch granules are formed through a complex packing process, with their chemical composition—including lipids, proteins, and moisture—varying depending on growth conditions and the source. Lipids, mainly phospholipids, remain from the amyloplast membrane on the starch surface. Additionally, triglycerides of fatty acids, such as linoleic, palmitic, and oleic acids, are present [27,29]. The total lipid content ranges from 0.014% to 3.04% across various starch sources (Table 1). High lipid concentrations are typically associated with B-type granules [30]. Lipids function as hydrophobic complexing agents that can remain within the AM helix through van der Waals forces. The stability of this complex is influenced by factors such as the nature of the lipid, the AM chain length, the solvent, and the processing conditions [31]. In addition, the lipid concentration is an important parameter because it forms a complex with starch, reducing the swelling power and starch solubility, which in turn affects starch gelatinization and retrogradation [32]. Researchers have reported the formation of a ternary starch–lipid–protein complex (Figure 1B), which modifies starch properties such as pasting, gelatinization, and rheological behavior [32,33]. Rheological measurements have determined that the complex forms during food processing, starting at 75 °C, and is significantly affected by protein content, shear rate, and temperature. Additionally, these complexes are stabilized by noncovalent interactions such as hydrogen bonds, hydrophobic interactions, and van der Waals attractions [33]. The starch–lipid–protein complex blocks the inner channels on the granule surface, affecting swelling and the accessibility of biological agents like enzymes. These channels are formed by the orderly non-reducing ends of AM and AP in a radial arrangement, oriented toward the center of the starch granule [34].

**Table 1:** Starch sources and their chemical composition.

Source	% Starch	% AM	Size Granule (μm)	% Protein	% Ash	% Lipids	% Fiber	Ref.
Cassava	93.17 ± 0.30	16.71 ± 1.00	17.24 ± 1.0	0.31 ± 0.01	0.23 ± 0.06	0.20 ± 0.00	0.23 ± 0.06	[35]
Potato	85.90–88.10	26.20–29.10	20.60–30.90	0.31–0.34	-	-	-	[36]
Rice	90.42	-	-	7.00 ± 0.06	1.81 ± 0.05	0.77 ± 0.15	-	[37]
Corn	9.00–23.00	-	-	10.00–13.00	2.00–5.00	2.00–3.00	12.30	[38]
Maize	-	29.30 ± 0.17	7.00–28.00	0.50 ± 0.00	0.38 ± 0.00	0.68 ± 0.02	-	[39]
Wheat	69.50	-	2.80–42.80	11.90	-	-	-	[40]
Chayote	-	12.90 ± 0.64	7.00–50.00	0.29 ± 0.00	0.46 ± 0.04	0.16 ± 0.00	-	[39]
Barley	72.20 ± 0.60	25.80 ± 0.70	16.30	13.50	-	3.04 ± 0.40	11.80 ± 1.00	[41]
Yam bean	-	-	-	1.23 ± 0.02	1.24 ± 0.03	1.17 ± 0.04	10.94 ± 0.02	[42]
Yam	99.70	22.20	28.50–30.60	0.06	0.13	0.03	0.11	[43]
Taro	96.75	19.37 ± 0.93		1.46 ± 0.10	0.98 ± 0.05	0.43 ± 0.01	0.39 ± 0.01	[44]
Taro	99.70 ± 0.40	8.40 ± 0.20	1.30–2.20	0.35 ± 0.00	0.28 ± 0.00	-	-	[45]
Canna	-	20.60	-	0.07	0.25	0.01	-	[46]
Mung bean	-	19.60	-	0.56	0.16	0.14	-	[46]
Sorghum	-	27.18 ± 9.98	-	0.31 ± 0.00	0.04 ± 0.00	0.05 ± 0.02	0.14 ± 0.03	[47]
Pejibaye fruit	79.00 ± 0.12	12.40 ± 0.18	-	0.54 ± 0.07	0.18 ± 0.07	0.93 ± 0.01	-	[48]
Banana	97.20 ± 2.40	-		2.03 ± 0.15	1.30 ± 0.30	-	-	[49]
Mango seeds	21.00	-	-	0.68	1.15	0.36	-	[50]
Chestnut	93.20 ± 1.10	24.70 ± 0.90	4.00–21.00	0.48 ± 0.02	-	-	-	[51]
Pinhão	-	22.25	-	0.07	0.08	1.00	-	[52]
Yambean (*Sphenostylis stenocarpa*)	-	34.40 ± 0.40	-	0.42 ± 0.01	0.25 ± 0.10	0.96 ± 0.10		[53]
Anchote	-	32.14 ± 0.19	-	-	-	-	-	[54]
Peach palm	71.00 ± 2.15	1.52 ± 0.04	-	0.47 ± 0.13	0.18 ± 0.02	0.55 ± 0.06	-	[55]
Breadfruit	98.86	27.68 ± 0.75	4.24–7.88	0.61 ± 0.01	0.47 ± 0.04	0.06 ± 0.01	-	[56]
Seed of loquat	93.78–54.31	45.69–6.22	29.05–43.66	0.61–1.86	0.39–0.28	0.80–0.41	-	[57]
Tomato	-	17.40–19.10	13.50–14.30	-	-	-	-	[58]
Ramon	92.57 ± 2.89	25.36 ± 2.37	3.00–26.00	-	-	-	1.15 ± 0.01	[59]
Unripe	99.31 ± 0.01	28.79 ± 0.10	27.30–42.00	0.36 ± 0.01	0.22 ± 0.01	0.08 ± 0.01	0.03 ± 0.01	[60]
Longan	84.40 ± 1.00	25.10 ± 0.30	1.57–7.66	0.08 ± 0.01	-	-	-	[61]
Loquat	85.70 ± 0.40	26.40 ± 1.10	5.21–9.36	0.12 ± 0.01	-	-	-	[61]
Myrosma cannifolia	438.60 (g kg^−1^)	225 (g kg^−1^)	8.00–17.50	12.64 (g kg^−1^)	5.48 (g kg^−1^)	2.51 (g kg^−1^)	0.07 (g kg^−1^)	[62]

## 3. SHs Formation Methods

**Figure 2:**
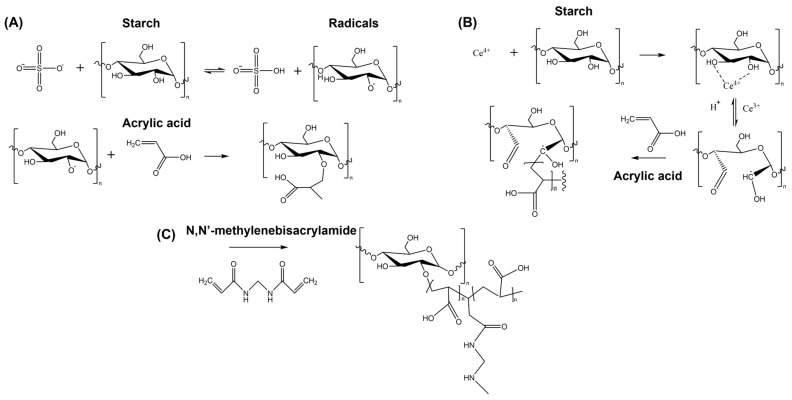
(**A**) Mechanism of starch-g-poly(acrylic acid) formation initiated by K persulfate. (**B**) Mechanism of starch-g-poly(acrylic acid) formation initiated by ceric (IV). (**C**) Starch-g-poly(acrylic acid) hydrogel formation using N,N′-methylenebisacrylamide as a crosslinking agent (modified from [64]).

SHs can be formed through (i) graft copolymerization of monomers onto the starch chains, (ii) using a chemical crosslinker, and (iii) by physical interactions between polysaccharide chains. Graft copolymerization of acrylic acid onto starch chains has been reported using ammonium persulfate (KPS) or ceric salts as polymerization initiators (Figure 2A,B). The persulfate ion acts upon heating, forming a sulfate anion-radical, which abstracts hydrogen from the starch hydroxyl groups, creating a radical on the polysaccharide chain. This provides an active center to initiate acrylic acid polymerization, resulting in the formation of the graft copolymer [63,64]. On the other hand, ceric (Ce^4+^) forms a redox complex with the hydroxyl groups of starch, which is then dissociated to generate a carbon radical on the starch chains, thereby initiating graft polymerization. Starch graft copolymerization using persulfate and ceric salts has also been reported with copolymers such as acrylamide [65], styrene [66], methyl methacrylate [67], acrylonitrile [68], and others. A review of experimental conditions for starch graft copolymerization was reported by Meimoun et al. (2017) [69]. In this section, we will focus on the characteristics and properties of the hydrogels obtained. The crosslinking material is formed after copolymerization, typically using crosslinking mediators like N,N′-methylenebisacrylamide (MBA) (Figure 2C). Czarnecka and Nowaczyk (2020) created a starch superabsorbent hydrogel with acrylic acid using ceric ammonium nitrate (CS-g-PAA/CAN) or KPS (CS-g-PAA/KPS) as the initiator [64].

**Figure 3:**
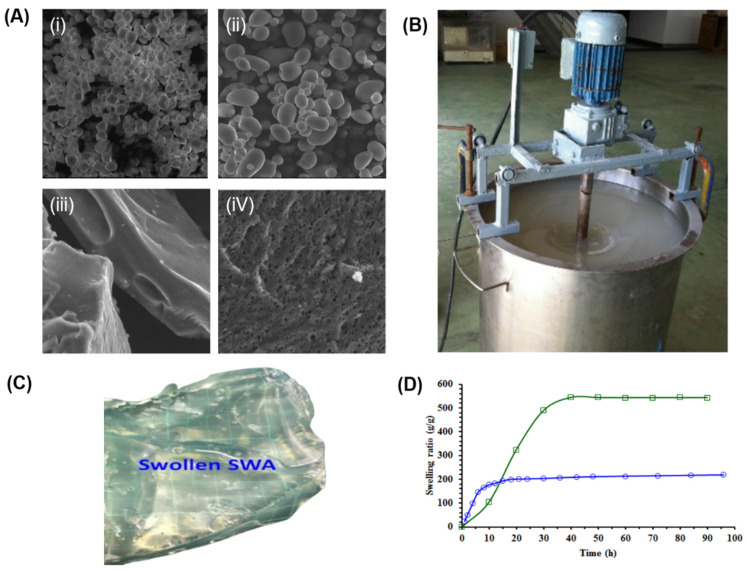
(**A**) SEM images at 1000× magnification of (i) corn starch, (ii) solvent starch, (iii) CS-g-PAA/CAN, and (iv) CS-g-PAA/KPS. (**B**) Up-scaled production of CS-g-PAA using a 150 L reactor. (**C**) Swollen CS-g-PAA hydrogel. (**D**) Swelling ratio of CS-g-PAA hydrogel for up-scaled production (blue line) and lab-scale synthesis (green line). Modified from (**A**) [64]; (**B**–**D**) [71].

Figure 3A shows the SEM images of corn starch and the formed hydrogels, highlighting differences in morphology and pore size. The CS-g-PAA/KPS hydrogel exhibits a porous surface, with pore sizes ranging from 11 to 136 μm, providing accessible spaces and channels that facilitate the hydration of hydrophilic groups, resulting in higher swelling. Another method for grafting starch with acrylic acid involves radiation. This technique is highly efficient and is initiated by hydroxyl radicals generated when water molecules are irradiated with gamma rays. The hydroxyl radicals remove hydrogen from the starch chain, thereby generating a polymer matrix [70].

Another widely studied hydrogel is starch-g-PAM (PAM stands for polyacrylamide), which is formed by the polymerization of the acrylamide monomer in the presence of an initiator such as KPS. Mu et al. (2022) reported the polymerization reaction using water as the solvent at 80 °C for 4 h, with the addition of MBA for crosslinking [72]. Starch grafting with acrylamide can also be achieved using KPS combined with microwave irradiation followed by cooling cycles. The grafting percentage increased from 71.25% to 1237.32% as the mass of acrylamide was increased from 2 to 10 g. A similar trend was observed, where increasing the irradiation time also led to an increase in the grafting percentage [73]. However, the properties of CS-g-PAA (using cassava starch in this experiment) were affected by both lab-scale and up-scaling production processes. Specifically, the degree of grafting (Dg) and swelling ratio (SW) were influenced, with a higher Dg observed in ups-caled production (using a 150 L reactor, Figure 3B). Consequently, this resulted in a lower SW compared to the lab-scale synthesis [71]. This can be observed by tracking the SW over time, with the burst profile for both materials reaching approximately 200 and 550 g/g for lab-scale synthesis and up-scaled production, respectively (Figure 3C,D). Gamma and electron beam radiation methods have also been applied to form other hydrogels, such as starch-g-PVA, at room temperature. Fourier Transform Infrared (FTIR) analysis suggested that the grafting reaction occurs predominantly on the AM chains. This hypothesis was supported by yield stress measurements, which showed a higher gel fraction in starch-g-PVA and AM-g-PVA hydrogels compared to the AP-g-PVA hydrogel system. Furthermore, an increase in starch concentration resulted in decreased swelling behavior due to the poor hydrophilicity of starch [74].

### 3.1. Crosslinking Chemical Methods

**Figure 4:**
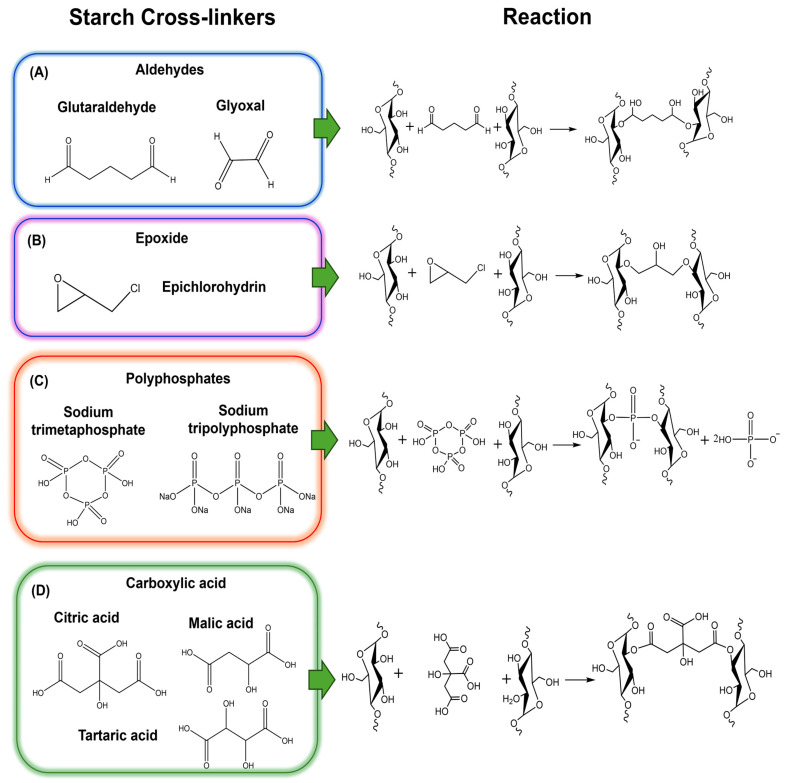
Common chemical crosslinkers used in the synthesis of SHs: (**A**) aldehydes, (**B**) epoxides, (**C**) polyphosphates, and (**D**) carboxylic acids. An example reaction is shown for each functional group.

AM and AP contain hydroxyl groups that can react covalently with crosslinkers such as glutaraldehyde (GA), epichlorohydrin (EH), sodium trimetaphosphate (STMP), and bi- or tricarboxylic acids (Figure 4) [75]. Corn starch was modified by crosslinking using GA [76]. The degree of substitution (DS) was found to be influenced by the pH of the reaction medium, increasing under alkaline conditions [76]. FTIR analysis revealed that in acidic environments, the hydroxyl band of the crosslinked starch shifted to higher frequencies, suggesting a decrease in hydrogen bonding. In contrast, this spectral shift was not observed when the crosslinking occurred in an alkaline medium [76]. GA has also been used in polymeric blends, such as starch/polyvinyl alcohol (PVA) hydrogel films [77]. The material formation involves three steps: (i) gelatinizing the starch at 70 °C for 1 h, (ii) mixing the PVA aqueous solution, gelatinized starch, and GA for 2 h, and (iii) drying the material at room temperature. The hydrogel swelled by approximately 250% in water and showed no dependency on pH, as both starch and PVA contain no ionizable functional groups. Recently, other research has reported the use of liquid ionic compounds, such as BMIM-BF4 (1-Butyl-3-methylimidazolium tetrafluoroborate), to enhance the plasticity and generate porosity within the hydrogel, increasing the swelling capacity (SC) up to 300% [78].

Another alternative for starch crosslinking is EH, an epoxide organochlorine that requires basic aqueous conditions, typically NaOH at 40 °C [79]. Studies have shown that the swelling power of EH-crosslinked starch decreases with increasing concentrations of both the crosslinker and the polymer. Furthermore, SC also diminishes with rising salt concentrations. This behavior is attributed to a reversal of polarity on the starch backbone, which elevates the ionic strength within the gel, creating osmotic pressure and limiting fluid infiltration. Additionally, this method can lead to imperfections in the gel due to micro-heterogeneities, which cause entanglements and micro-aggregations of the crosslinker within the hydrogel network [79]. EH has also been used to crosslink starch in the presence of humic acid [80], following the methodology proposed by Kulicke et al. (1989) [79], thereby enhancing the material’s properties and expanding the range of starch applications. On the other hand, starch can be phosphorylated through reaction with inorganic phosphate compounds, such as STMP, forming covalent phosphate diester bonds that act as crosslinks within the starch network [81]. Approximately 60–70% of the total P is bound to the C-6 position of the starch anhydroglucose units, with the remainder attached at the C-3 position. The rheological behavior of these hydrogels depends on both temperature and NaOH concentration, as these factors influence the stability of the diester phosphate bonds. An increase in NaOH concentration promotes hydrolysis of the diester linkages, reducing viscosity by disrupting the entanglement points within the gel matrix.

The crosslinking of corn starch with STMP has also been reported by Seker and Hanna (2006) using reactive extrusion (REX) at 130 °C [82]. The crosslinking mechanism involves the ring opening of STMP, followed by radical grafting to form starch tripolyphosphate (TPP). The reaction is completed when the phosphate end group reacts with another starch polymer, resulting in a covalent crosslinked bond [83]. STMP has also been applied in the (REX) of starch–carboxymethyl (SCM) cellulose to form hydrogel films at different pH levels. These films demonstrated higher swelling capacities compared to pure starch films. Moreover, the grafted carboxymethyl (CM) cellulose improved the mechanical properties of the resulting hydrogels. At higher pH values, the ionization of carboxyl and phosphate groups further increased the swelling degree due to enhanced electrostatic repulsion [83]. A mixture of STMP and sodium TPP has been used to crosslink various starches, including potato, banana, corn, cassava, and breadfruit. According to Lemos et al. (2020) [84], the introduction of hydrophilic phosphate groups via phosphate diester covalent bonds increases repulsion between phosphate units, thereby reducing the intermolecular forces between starch chains. This structural modification allows for greater water accommodation and uptake. As a result, the enhanced electrostatic repulsion also facilitates water percolation and absorption throughout the starch matrices [85].

Organic acids such as citric acid (CA), malic acid (MA), and tartaric acid have been explored as crosslinking agents for starches. Their incorporation into the polymeric matrix enhances mechanical resistance and reduces permeability. Additionally, these acids contribute to starch hydrolysis, facilitating granule disruption and promoting the formation of a more homogeneous matrix [86]. For example, Simões et al. (2019) developed starch/xanthan gum hydrogels using CA as a crosslinker [87]. Beyond its crosslinking function, CA also exhibits hydrolytic and plasticizing properties. The resulting materials displayed a smooth surface with a slightly yellowish hue, a characteristic commonly reported for starch CA composites [88]. The SC of these hydrogels depends on the proportion of CA used during material formation, which also influences mechanical properties particularly by increasing elongation due to CA’s hydrolytic action. CA has also been used to crosslink starch in the presence of glycerol at 75 °C. Thermal analysis suggests that CA initially reacts with glycerol, followed by the formation of a starch–glycerol–CA complex [89]. Another organic acid, MA, has been evaluated as a crosslinking agent in interpenetrating polymer network hydrogels composed of natural rubber latex (NR) and cassava starch [90]. Tensile strength was found to decrease as starch content decreased. Moreover, starch plays a crucial role in the material’s swelling behavior. After 100 h of water contact, the hydroxyl groups in starch facilitate the absorption of a significant amount of water molecules, leading to polymer chain expansion and enabling the material to retain a large volume of water.

MA has also been applied to crosslink sago starch with PVA [91], revealing that the swelling behavior of sago starch–maleate–PVA hydrogels depends on precursor composition, pH, and the temperature of the swelling medium. The SW increased as the temperature rose from 25 °C to 37 °C, which was attributed to polymer chain expansion at higher temperatures. This expansion enhances the diffusion rate of water molecules into the gel and facilitates their entrapment within the hydrogel network. In addition, pH was shown to influence SC by affecting both the pore volume and water diffusion through the material. The SW was lower at a medium pH of 1.23 (similar to gastric juice) than at pH 6.38 (intestinal fluids) or pH 7.0. This behavior is attributed to the acidic nature of the hydrogel, which contains free carboxyl groups on the polymeric starch chains. At higher pH values, stronger interactions between the hydrogel and the surrounding medium enhance swelling. Furthermore, crosslinking with di- and tricarboxylic acids affects the SC, as observed in starch phosphate hydrogels crosslinked with CA, MA, succinic acid, glutaric acid, and adipic acid, which exhibited swelling capacities ranging from 150 to 185 g/g [92]. However, the starch source also significantly influences hydrogel swelling. This was evident in hydrogels made from waxy maize starch, high-AM starch, degraded starch, and potato starch crosslinked with CA. The highest SC was observed in hydrogels made from degraded starch, likely due to its lower molar mass, which improves accessibility to crosslinking agents and water.

Glyoxal (GYX), a dialdehyde, facilitates the formation of starch–κ-carrageenan hydrogels through hemiacetal bonds, resulting in a stable crosslinking structure at room temperature. The methodology involves two steps: (i) dispersing starch and κ-carrageenan separately in a 10% (*v*/*v*) GYX solution and heating each to 80 °C for 20 min, and (ii) mixing the polymer dispersions at 80 °C. This process exemplifies a simple and scalable method for hydrogel preparation, making it suitable for industrial applications such as plant nutrient delivery [93]. Starch can also be chemically modified to enable alternative crosslinking mechanisms, such as thiol-ene click chemistry between starch chains. However, this method involves multiple synthesis steps compared to more conventional approaches. The process includes (i) synthesizing allylic starch in a micellar medium using allyl chloride for 24 h at 45 °C under agitation [94]; (ii) producing carboxyl succinic starch (St-COOH) via esterification in DMSO at 70 °C; (iii) obtaining thiolated starch (St-SH) by coupling L-cysteine hydrochloride to the carboxylic acid groups of St-COOH; and (iv) forming SHs by mixing AS and St-SH in PBS, followed by UV irradiation (365 nm, 36 W) for 30 min. The resulting hydrogel demonstrated high biodegradability when exposed to β-amylase at pH 6.7–7.0, which efficiently degraded the starch chains. The development of starch-based polymeric blends also broadens the spectrum of crosslinkers that can be utilized in hydrogel formation. For instance, an alginate/SH hydrogel was synthesized using crosslinkers such as MnCl_2_, ZnCl_2_, and CaCl_2_. In this system, Mn^2+^, Zn^2+^, and Ca^2+^ ions crosslink alginate while simultaneously entrapping starch within the polymeric matrix, thereby increasing the material’s hydrophobic domains [95]. A similar strategy was applied to produce a polymeric hydrogel from starch and CS, a natural polycationic polymer known for its biocompatibility, biodegradability, and non-toxicity. However, the high cost of CS limits its widespread industrial application. Combining it with the more economical biopolymer starch enhances the composite material’s performance while reducing production costs, making it viable for various industrial processes [96]. In this case, TPP acts as a crosslinker by forming ionic interactions between its negatively charged phosphate groups and the protonated amino groups of CS, effectively entrapping starch within the matrix. This hydrogel system showed great potential for the controlled release of plant growth-promoting bacteria (*Azospirillum brasilense* Az39 and *Pseudomonas fluorescens* ZME4). The polymeric matrix successfully released both bacterial strains into natural soils, demonstrating its suitability for agricultural applications [97].

### 3.2. Crosslinking Physical Methods

SHs crosslinked by physical methods are reversible and can be modified by environmental conditions [98]. These materials rely on non-covalent interactions such as hydrophobic forces, polyelectrolyte complexation (PEC), electrostatic interactions, and hydrogen bonding [99], thereby reducing the reliance on toxic crosslinkers typically used in chemical methods. However, while physically crosslinked SHs exhibit water insolubility, their overall stability is generally lower compared to those formed via chemical crosslinking [100]. In aqueous systems, starch granules are readily disrupted by heat, initiating the gelatinization process and enabling polymeric interactions with other components. Upon cooling (within a range of 4–75 °C), starch undergoes retrogradation, during which the polymer chains realign and recrystallize, forming a structured material within a mold.

Alternatively, SHs can be prepared in organic alcohols, resulting in the formation of alcogels. When subjected to freeze-drying to remove water vapor, the resulting material is termed a cryogel, characterized by a nanoporous structure with a high surface area [101]. However, this structural behavior is highly dependent on the starch source and the AM-to-AP ratio [102]. Such behavior is also observed in chemically modified starches. For instance, polyvinyl acetate (PVA) has been grafted onto starch via radical copolymerization, forming a hydrogel crosslinked by physical interactions. In this case, the SC of the starch-g-PVA hydrogel can be modulated by adjusting the length of the grafted PVA branches [103]. The crosslinking occurs primarily through hydrogen bonding between hydroxyl (–OH) groups, as seen in hydroxyethyl starch (HES)–PVA hydrogels, where FTIR analysis revealed that the physicochemical properties depend on the polymer ratios [104]. The hydrogel properties were strongly influenced by HES concentration, which affected both pore size and pore area distribution. In another example, CS—a natural polysaccharide—forms a stable polymeric blend with HES. The miscibility of this blend is attributed to hydrogen bonding between the reactive groups of CS and hydroxyethyl starch, resulting in improved compatibility and stability [105].

PEC hydrogels represent another class of physically crosslinked materials formed through ionic interactions. These hydrogels are created by the electrostatic attraction between oppositely charged polyelectrolyte chains, resulting in materials with notable swelling capabilities. Prado et al. (2009) developed PEC hydrogels using κ-carrageenan as the counter-polyion to starch modified with 2-hydroxy-3-(N,N,N-trimethylammonium) propyl groups, which confer a polycationic character [106]. Swelling tests showed that the hydrogel maintained structural integrity, exhibiting rapid swelling within the first hour, reaching an SC of 380%. To further enhance the performance of PEC SHs, starch was modified with CM groups, enabling complex formation with CS. The resulting PEC from starch–carboxymethyl (SCM) and CS exhibited a prolonged disintegration time, indicating improved stability [107]. However, the swelling behavior of the SCM–CS hydrogel depends on the properties of the swelling medium. At pH 1.2, the hydrogel demonstrated greater swelling compared to pure water. This enhanced swelling at an acidic pH is attributed to the protonation of functional groups within the polymeric matrix, which promotes electrostatic repulsion and facilitates water absorption [108].

## 4. Application of SHs to Transport Fertilizers

The applicability of SHs for nutrient delivery in soils and plants depends on several key material properties, including encapsulation efficiency and SC, which directly influence nutrient diffusion and the chemical interactions responsible for nutrient retention. Moreover, the nutrient release process is significantly affected by environmental factors such as pH, soil characteristics, ionic strength, and crop type, among others. This section is structured as follows: (i) fertilizer encapsulation and in vitro assays, and (ii) fertilizer release in soils and plants.

### 4.1. Fertilizers Encapsulation and In Vitro Assays

SHs (starch-based hydrogels) have been applied for the encapsulation of essential macronutrients such as N, P, and K. The nutrient release profiles from these hydrogels have been evaluated in aqueous media, with release times and kinetic modeling results as summarized in Table 2. Hydrogels represent an innovative platform for developing slow-release fertilizers, where nutrient encapsulation typically occurs during the hydrogel formation process. For instance, A. Sofyane et al. (2021) encapsulated P and N from diammonium phosphate fertilizer into starch acetate/PVA/glycerol hydrogels [109]. The hydrogel was prepared by mixing the polymers and glycerol in an aqueous solution at 90 °C, followed by the addition of the fertilizer under continuous stirring for 30 min. The resulting mixture was then poured into a Teflon mold and dried at room temperature. Complete release (100%) of the encapsulated P and N occurred within the first 100 min.

Alternatively, nutrient encapsulation can be performed post-synthesis via adsorption. For example, CS/SHs hydrogels crosslinked with TPP have been used for the controlled release of KNO_3_ [110]. The hydrogels were formed using different mass ratios of CS and potato starch by dispersing the polymers in lactic acid, followed by crosslinking with TPP. Hydrogel beads were then produced using a dripping technique. Nutrient loading was achieved by immersing the dried beads in a 20% (*w*/*v*) KNO_3_ solution for 4 h. These hydrogels exhibited a slow-release profile, releasing 25–35% of the nutrient within 5 h, depending on polymer composition, and reaching approximately 94% release after 16 days. A similar methodology was reported by [111] León et al. (2019), who encapsulated urea, KNO_3_, and ammonium sulfate ((NH_4_)_2_SO_4_) in hydrogels prepared from starch oxidized with KMnO_4_/NaHSO_3_. Various starch sources were evaluated, including corn, sweet cassava, and bitter cassava [111]. The water uptake capacity of the hydrogels increased under basic pH conditions due to the ionization of carboxylic groups formed during starch oxidation, enhancing swelling and nutrient diffusion [111].

Hydrogels immersed in nutrient solutions exhibited varying encapsulation efficiencies: urea and KNO_3_ showed relatively low values (30% and 12%, respectively), whereas ((NH_4_)_2_SO_4_) demonstrated a high encapsulation capacity of 90%. According to the release profiles, all hydrogels released more than 50% of the encapsulated nutrients within the first 4 h. This rapid release is attributed to the quick dissolution of the nutrients during the initial swelling phase. Therefore, achieving effective controlled nutrient release remains a significant challenge. To address this, hydrogels based on high-amylose (AM) maize starch oxidized with KMnO_4_/NaHSO_3_ were grafted with maleic acid in the presence of sorbitan monooleate and methylene bisacrylamide (MBA) to improve the absorption and controlled release of K-based fertilizers [4]. The oxidation of starch hydroxyl groups at the C-2, C-3, and C-6 positions leads to the formation of aldehyde and carboxyl functionalities. Grafting of disodium maleate, monosodium maleate, and MBA onto the macroradicals produced results in self-crosslinking of the starch chains and the introduction of various functional groups. The hydrogel showed an adsorption capacity of 50% to 90% for KH_2_PO_4_, a common source of K^+^ and PO_4_^3−^. Regarding nutrient release, between 20% and 50% was released within the first 8 h in water, followed by a gradual release over time, reaching approximately 60% after 300 h (12.5 days). Notably, less than 70% of the total nutrient content was released, indicating that a portion remained entrapped within the polymeric matrix.

**Figure 5:**
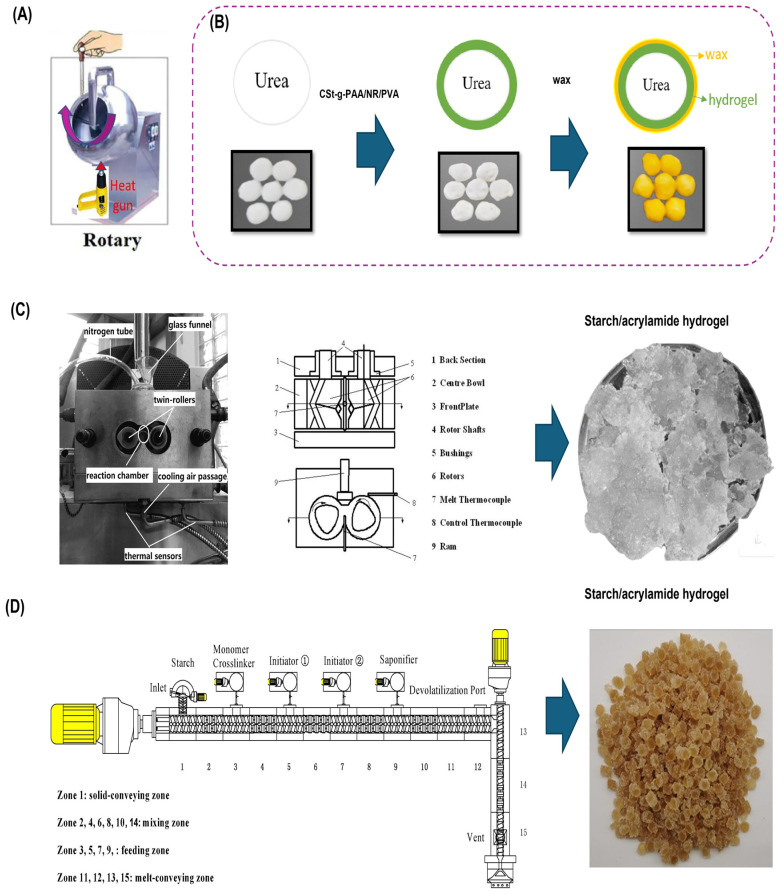
(**A**) Rotating drum machine equipped with a hot-air gun used for CSt-g-PAA/NR/PVA coating. (**B**) Sequence of coating urea with CSt-g-PAA/NR/PVA hydrogel and wax. (**C**) HAAKE Rhemix 600p twin-rotor mixer used for starch/acrylamide hydrogel production. (**D**) Twin-screw extruder connected to a single extruder with four injection ports for starch/acrylamide hydrogel production. Modified from (**A**,**B**) [112]; (**C**) [114]; (**D**) [115].

Machines are employed to coat nutrients with hydrogels, enabling the large-scale production of controlled-release fertilizer systems. For instance, a rotating drum machine equipped with a hot-air gun (Figure 5A) was used to coat urea prills with a cassava starch-g-polyacrylic acid/NR/PVA (CSt-g-PAA/NR/PVA) hydrogel composite [112]. The hydrogel dispersion was slowly sprayed onto the surface of the rotating urea prills (at 35 rpm), resulting in a uniform biodegradable hydrogel coating. Subsequently, the coated prills were covered with a wax solution (Figure 5B) to enhance water resistance and further control nutrient release. The hydrogel-encapsulated urea exhibited a sustained release profile, delivering approximately 80% of the nutrient over the first 80 h. After this period, the cumulative release plateaued and remained constant for the duration of the experiment (160 h). To mathematically describe and predict the release behavior of nutrients from such systems, several kinetic models are commonly employed, including zero-order, first-order, Higuchi, and Korsmeyer–Peppas models. The urea release from the CSt-g-PAA/NR/PVA hydrogel followed the Korsmeyer–Peppas model, indicating that the release mechanism is governed by Fickian diffusion.

Another machine employed in the production of SHs is the hot-compression vulcanizer, which was used to synthesize slab-shaped, urea-embedded grafted SHs. In this process, corn starch, acrylamide, water, and urea were combined in a Haake mixer. The protocol involved gelatinizing the starch at 80 °C for 10 min, followed by a cooldown to 30 °C to prevent premature chemical reactions [113]. The urea release profile of these SHs in water exhibited an initial burst, with approximately 50% of the nutrient released within the first 24 h. This was followed by a cumulative release reaching 78% over a 40-day period. Notably, increasing the starch concentration reduced the hydrogel’s ability to sustain urea release, as higher starch content diminished the initial burst. This release behavior could be adjusted by modifying the acrylamide-to-starch ratio; increasing the corn starch proportion effectively decreased the burst effect. The overall release kinetics conformed to a zero-order model, suggesting a dual mechanism involving Fickian diffusion and matrix erosion resulting from polymer degradation.

A HAAKE Rheocord Polylab RC500p system, equipped with a HAAKE Rhemix 600p twin-rotor mixer (Figure 5C), was employed to produce starch/acrylamide-based hydrogels by crosslinking with N,N′-methylenebisacrylamide for urea encapsulation [114]. The chemical modification process involved adding the reagents to the mixer at 80 °C, after which the temperature was reduced to 65 °C by air and N injection. The crosslinking reaction was initiated in the presence of ceric ammonium nitrate as an oxidizing agent. Urea was subsequently added to the reaction mixture and blended for 40 min at 80 °C and 80 rpm to form the SHs. The resulting material demonstrated high efficiency for slow urea release in static water, with only 15% of the nutrient released within the first 5 days. Between days 5 and 20, more than 50% was released, reaching over 80% by day 40. This approach presents a simple and cost-effective technique for the controlled delivery of nutrients using functional polymeric matrices

**Table 2:** Starch material applied to deliver nutrients (in vitro assays).

Material	Nutrient	Observation	Reference
Hydrogel: Sulfonated corn starch/poly(acrylic acid) synthesized by polymerization	P	P exhibited a sustained release over 72 h; however, 40% of the entrapped P was released within the first 24 days.	[116]
Hydrogel: PVA-modified starch biopolymer crosslinked with CA	N	The transport of nutrients through the swollen network of macromolecular polymer chains follows a non-Fickian or anomalous diffusion mechanism.	[117]
CM starch-graft-polyacrylamide (PCMS-g-PAM)	P	The phosphate release mechanism from P-CMS-g-PAM was analyzed using the Korsmeyer–Peppas model.	[118]
Starch acrylamide, N,N′-methylene-bisacrylamide (crosslinker) by hot compression vulcanizer	N	The results showed that a higher gel strength was achieved with lower urea loading, increased grafting content, and greater crosslinking density in starch-based hydrogels. Additionally, the hydrogel network exhibited zero-order, or at least time-independent, urea release kinetics during the intermediate stable stage.	[113]
Starch acetate (SA)/PVA/glycerol (GLY)	N, P	The time required to reach the maximum concentration of N release was 2.4 and 3.2 times longer than that of uncoated DAP when DAP was coated with CF-3 (70% SA) in a single layer and double layer, respectively, indicating improved slow-release properties for N.	[109]
Cassava SHs crosslinked with CA	N, K, P	The hydrogel showed a slow release of the nutrient to P, releasing up to 30% in 150 h; however, N and K showed a burst release effect in the first 15 h, releasing until 80% of the nutrient was entrapped in the polymeric matrix. Additionally, the nutrient release followed a Fickian diffusion from the hydrogel to the bulk of the diffusion medium.	[119,120]
Maize starch (AS) was modified by graft copolymerization with sodium acid maleate and disodium maleate and covalently crosslinked with N,N′-bismethylene acrylamide	K, P	The fertilizer loading percentages for KNO_3_ ranged from 95% to 57%, while for KH_2_PO_4_, they ranged from 90% to 55%. The release percentages were between 92% and 67% for KNO_3_ and between 89% and 61% for KH_2_PO_4_, respectively.	[4]
Starch-g-poly (acrylic acid-acrylamide)/\zeolite hydrogel composite	N, P, K	Measuring the concentrations of N, P, and K released in buffer solutions at pH 5, pH 7, and in water over a period of 0 to 100 h revealed a gradual release pattern.	[121]
Hydrogel formed by starch oxidation with KMO_4_ and NaHSO_4_	N, K	The loading and release of the fertilizer depended on the initial fertilizer concentration in the medium, as well as the nature, structure, and morphology of the starch used.	[111]
Hydrogel formed by the starch (corn, sweet cassava and bitter cassava) oxidation with KMO_4_ and NaHSO_4_	N, K	The SC of the oxidized samples follows the trend of Na_2_SO_4_ > NaCl > KNO_3_ > CaCl_2_. All starch samples exhibit a similar “S”-shaped release kinetic profile, consisting of three main stages. A noticeable “burst effect” was observed up to 0.5 h, particularly for KNO_3_.	[122]
CS/starch hydrogel crosslinking with sodium TPP	P, N	The fertilizer release ratio exceeded 70% of the total loading. The hydrogel composition and crosslinking time determined the mechanism governing K release, which was controlled by matrix relaxation.	[110]
Hydrogel formed by potato starch, acrylic acid, acrylamide, and β-cyclodextrin modified by maleic anhydride, accompanying halloysite nanotubes	N	The results suggested that the prepared fertilizer exhibited excellent water retention and urea release control. Moreover, the addition of halloysite enhanced the fertilizer’s release properties. The study on urea release kinetics indicated that urea release in water was controlled by its concentration, whereas its release in soil followed the Fickian diffusion mechanism.	[123]
Starch-based superabsorbent hydrogels reinforced with natural char nano/microparticles	N	Starch-based superabsorbent nanocomposites exhibited a Fickian water diffusion mechanism and followed a pseudo-second-order swelling kinetic model. Moreover, after 14 days, the NCNP/hydrogel nanocomposite demonstrated a threefold increase in water retention capacity compared to the neat hydrogel (23.1% vs. 7.1%, respectively).	[124]
Starch-based superabsorbent polymers (SBSAPs) using as initiator (ceric ammonium nitrate, or CAN) and crosslinker (N,N0-methylene-bisacrylamide)	N	More than 50% of the urea was released at a significantly higher rate. Subsequently, between 20 and 40 days, over 80% of the urea was released.	[114]

The use of machinery enables the large-scale production of SHs. One promising method is REX, a continuous, solvent-free process in which polymer reactions occur under high-viscosity conditions within an extruder. This technique is both cost-effective and environmentally friendly [125]. Jiang et al. (2022) developed a twin-screw extruder system connected to a single extruder equipped with four injection ports, allowing the separate feeding of monomers, initiator-1 (ceric ammonium nitrate), initiator-2 (KPS), and a saponifying agent (sodium hydroxide) for the graft copolymerization of acrylamide onto corn starch [115]. A schematic representation of this REX system is shown in Figure 5D. The process was conducted at a constant rotation speed of 90 rpm across all reaction zones. The crosslinker was first injected via a metering pump in zone 3, followed by the sequential injection of the initiators in zones 5 and 7, and finally, the saponification agent in zone 9. The reaction mixture underwent thorough mixing and devolatilization through ports located between zones 13 and 15 to remove volatile byproducts. FTIR and NMR analyses confirmed the success of the grafting reaction, with a monomer conversion rate of 97%. This system demonstrates strong potential for the industrial-scale fabrication of SHs designed for controlled fertilizer release.

Nanoparticles (Nps) and microparticles have demonstrated great potential as modifiers of hydrogel properties when incorporated into the polymeric matrix. This strategy enhances the interactions between the matrix and the encapsulated nutrient, thereby improving the nutrient release profile. For example, natural charcoal Nps (NCNPs), with particle sizes below 25 nm, were incorporated into a starch-g-poly(acrylic acid-co-acrylamide) [starch-g-poly(AA-co-AAm)] superabsorbent hydrogel to enhance its performance in controlled slow-release applications [126]. FTIR analysis revealed that NCNPs contain oxygenated functional groups, which promote their stabilization within the polymeric network through hydrogen bonding and electrostatic interactions. The inclusion of NCNPs significantly altered the release behavior of urea, reducing the cumulative release from 80% to just 10% over a 21-day period. This sustained release follows a Fickian diffusion mechanism, highlighting the effectiveness of nanoparticle incorporation in controlling nutrient delivery.

The NCNPs promote interfacial polymer–filler interactions, which help extend the urea release time. This material has also been produced using a planetary ball-mill machine (NARYA-MPM), incorporating stearic acid crystals and zirconia particles with sizes ranging from 5 to 10 nm [124]. Clays have also been used in the formation of starch-g-poly(acrylic acid) hydrogels, such as mordenite, a natural zeolite (crystalline aluminosilicate) with siloxane and hydroxyl groups, which enable physical interactions with the polymer chains [127]. However, this material exhibited a high urea release rate, with 93% of the urea released within 48 h. Encapsulating the nutrient in nanomaterials has been another approach explored to reduce the release rate. For example, halloysite nanotubes modified with β-cyclodextrin and maleic anhydride were used to encapsulate urea and disperse it in SHs formed with potato starch, acrylic acid, and acrylamide [123]. The halloysite was treated with sulfuric acid to enlarge the volume diameters and increase the urea retention capacity. Additionally, β-cyclodextrin was used to encapsulate urea in its inner cavity, which allows it to encapsulate various compounds. The cumulative release of urea at 2 h, 6 h, and 12 h was approximately 50.9%, 66.7%, and 86.8%, respectively. This release rate was slower compared to urea without encapsulation (98% released within 2 h). The release behavior followed the Korsmeyer–Peppas model, suggesting Fickian diffusion from the pores and channels of the hydrogel. Furthermore, pH plays a role in nutrient delivery. Sjaifullah et al. (2020) studied the effect of pH on K release from a starch-g-poly(acrylic acid)/zeolite hydrogel composite [121]. The amount of K released was quantified using an ICP instrument. At pH 7, the hydrogel composite released K faster compared to pH 5.

### 4.2. In Vitro Evaluation of SHs in Soil and Plant System

**Figure 6:**
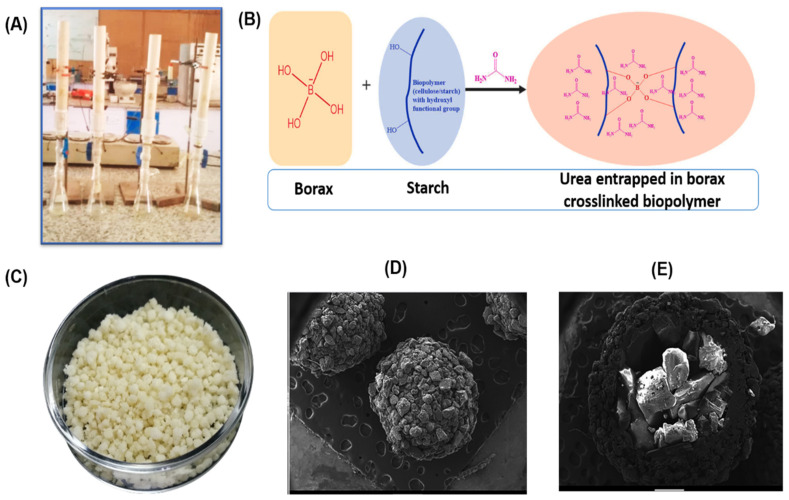
(**A**) PVC column used to evaluate nutrient release in soils. (**B**) Crosslinking mechanism between borax and starch to form a hydrogel. (**C**) Granules of urea coated with starch-g-poly(acrylonitrile) hydrogel. (**D**,**E**) Scanning electron micrographs of hydrogel-coated urea and a cross-section of the coated urea. Modified from (**A**,**B**) [129]; (**C**–**E**) [130].

Table 3 presents SHs and composites applied to nutrient delivery in soils and plants, respectively. The nutrient release depends on environmental conditions. Qiao et al. (2016) achieved a reduction in N application rates by coating urea granules with starch-g-polyacrylamide hydrogels formed using a twin-roll mixer with ceric ammonium nitrate as the initiator and MBA as the crosslinker [128]. The study evaluated the effect of the starch source (maize, potato, and cassava) on urea delivery in soil. After approximately 20 h, all materials delivered more than 40% of the urea encapsulated, with the hydrogel formed from potato starch showing a slow release rate (70% at 96 h). Nutrient release in soils was studied using a PVC column with a diameter of 5 cm and a length of 35 cm (Figure 6A). At the bottom of the column, a controlled tap was installed to collect soil leachate samples at various intervals. Typically, the soil is wetted to 100% of its water retention capacity [129].

The SH formed with borax was evaluated for controlling the delivery of urea in sandy loam soil. Figure 6B illustrates the potential formation mechanism of the hydrogels, with borax acting as a crosslinker. The chemical gelation of borax with the hydroxyl groups of the polysaccharide was observed through the B–O stretching vibration in the FTIR spectra at 1015 cm^−1^. The release rate of pure urea (without encapsulation) in soil reached approximately 96% within three days. In contrast, the hydrogel tested delivered about 14% of the urea in 6 days, with the maximum release occurring at 36 days, reaching approximately 94% of the encapsulated urea. This demonstrated that the hydrogel reduced the release time by 89% compared to in vitro release experiments in distilled water. Soil nutrient release experiments can also be performed in plastic beakers, using laterite sandy clay loam soil as a sample for each interval. Starch-g-poly(acrylonitrile) granule hydrogel (Figure 6C) showed lower N release (45%) after 7 days of incubation in laterite sandy clay loam soil compared with the control (79% in 7 days) [130]. The N release was extended up to 108 days, with approximately 70% of the urea released. According to the research, the urea release depends on water penetrating the coating, which dissolves the urea in the core (Figure 6D,E), allowing the urea to diffuse through the coating into the environment. On the other hand, water is essential for plant growth, but it can easily evaporate, decreasing the efficiency of plant growth. SHs with fertilizer can also be used to deliver water, improve soil aeration, reduce evapotranspiration, and prolong water availability for seeds and plants. To efficiently deliver nutrients to seeds, the coating material is usually applied via the dry coating method, which involves mixing the corn seeds with the wet hydrogel [131]. The effect of different concentrations of the material can be studied by mixing it with other components. For instance, bentonite was mixed with SH made from potato starch modified with succinic anhydride (starch–MS) to create a powder mixture with hydrogel concentrations ranging from 10% to 50% (*w*/*w*). Methylcellulose is commonly used as an adhesive in the solution to enhance the hydrogel-seed coating. Increasing the starch-MS concentration in corn seed coatings reduces the coating efficiency from 80% to 60%. This behavior can be attributed to the tendency of the MS to wick the adhesive solution, reducing the seed’s surface bonding with the powder material. The material was tested for seed growth in soil, but no significant differences in root and shoot dry weight were observed compared to the control (clay/talc). Starch-poly(sodium acrylate-co-acrylamide) superabsorbent hydrogel was tested in sandy soils with maize (*Zea mays*) seedlings [132]. The hydrogel improved the soil’s ability to retain water, enhanced aeration, promoted faster growth, and minimized the risk of root rot. Additionally, the material increased the cation exchange capacity in the soil by reducing cation leaching from the topsoil, creating a water- and nutrient-rich environment favorable to seed development. Chen et al. (2004) tested the water delivery of starch-g-polyacrylic acid hydrogels during corn seed germination, finding that the hydrogel had excellent water retention capabilities, promoting corn seed growth with a germination rate of 98% [133]. This suggests that the hydrogel has great potential for use in arid and desert regions to support plant growth.

**Figure 7:**
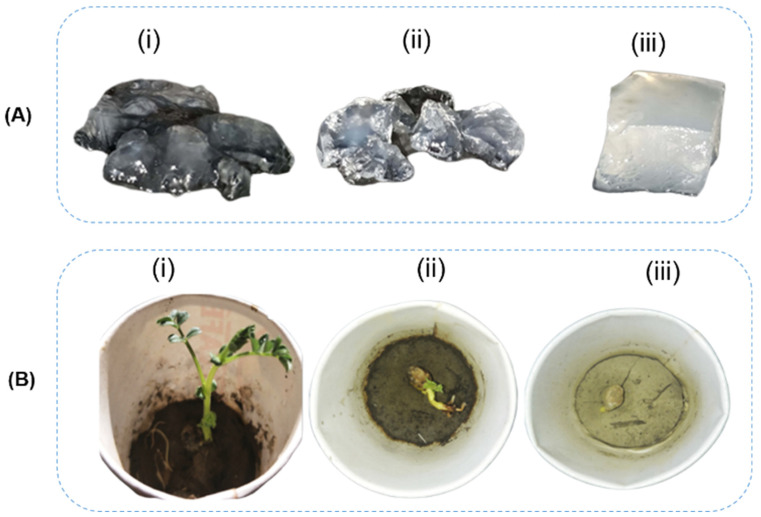
(**A**) Crosslinking density and strength of starch-modified poly(acrylic acid) (SAH) at (i) 43.57%, (ii) 50.97%, and (iii) 67.52% of acrylic acid. (**B**) SAH applied in soils: (i) soil with urea-encapsulated SAH, (ii) soil with SAH, and (iii) bare soil. Modified from [134].

The SH modified with acrylic acid was synthesized through a free-radical reaction using KPS as the initiator and MBA as the crosslinking agent [134]. After polymerization, the material was neutralized with NaOH, replacing the H^+^ ions of the carboxylic acid groups in the poly(acrylic acid) with Na+ ions, ensuring overall electrical neutrality within the polymeric chain. The concentration of the crosslinker plays a crucial role in the hydrogel’s properties, affecting both the swelling behavior and its visual appearance (Figure 7A). As the crosslinker concentration increases, the swelling ability of the hydrogel decreases due to the reduced distance between crosslinked points, leading to a more compact material. The biodegradation of the hydrogel was tested with bacterial strains Pseudomonas aeruginosa and Bacillus subtilis. The starch-based hydrogel degraded into small fragments over 6 weeks of incubation with both bacterial strains. The hydrogel demonstrated a high urea encapsulation efficiency (99.7%) and exhibited a slow-release profile. In the first 5 days, approximately 25% of the encapsulated urea was released, and between 20 and 30 days, nearly all of the urea (99%) was delivered. The release mechanism is attributed to diffusion through the polymeric matrix driven by the osmotic pressure difference between the interior and exterior of the gel. Urea release from the hydrogel significantly enhanced plant growth in chickpeas, increasing shoot length to 2.8 cm, compared to 0.5 cm in untreated soil. Additionally, the hydrogel alone (without urea) created an environment conducive to seed germination and plant growth (Figure 7B).

**Table 3:** SH applied to deliver nutrients in plants.

Material	Nutrient	Plant and Seeds	Observation	Reference
Natural rubber and cassava starch crosslinking with sulfur and GA	Urea	Corn and basil plants	The performance of encapsulated urea beads in corn and basil plantations was significantly higher compared to non-encapsulated urea. The material releases urea through the porous membrane via non-Fickian diffusion.	[135]
Starch phosphate carbamate hydrogel (SPC-hydrogel)	Urea	Maize seeds	The hydrogel exhibits a continuous slow-release performance, with 50.3% of urea released within 15 h and nearly complete release in just over 25 h in water.	[136]
Cassava starch-g-polyacrylic acid/natural rubber/PVA	Urea	Chili plant	The formulation demonstrated excellent slow-release N delivery in both water (47.5% at 168 h) and soil (38.5% at 30 days). The chili plant growth was effectively enhanced, with a production cost 4–5 times lower than that of commercial slow-release fertilizers.	[112]
Succinate-modified potato starch	Water	Corn seed	At a water supply of 77% field capacity, the coated seeds exhibited a significantly higher emergence rate compared to uncoated seeds.	[131]
Starch-g-poly(styrene-co-butylacrylate) nanocomposite	Urea	Tomato	An increase in the total N percentage and a decrease in nitrate content in the aerial parts of plants were observed compared to traditional urea.	[137]
Starch-modified poly(acrylic acid with N, MBA as crosslinker)	Urea	Chickpea plant	Seeds placed in soil treated with urea-encapsulated hydrogel exhibited a shoot length of 2.8 cm, whereas those in untreated soil had a shoot length of only 0.5 cm.	[134]
Starch-poly (sodium acrylate-co-acrylamide)	Water	Maize (*Zea may*) seed	The soil-hydrogel analysis conducted at monthly intervals revealed a significant improvement in soil moisture retention and enhanced growth performance of maize seedlings compared to the control.	[132]
Nanozeolite– CS/sago starch-based biopolymer composite	Urea	*Philodendron* sp. plant	The hydrogel significantly promoted the growth of *Philodendron* sp., leading to improved growth indices, including survival rate, number of leaves, leaf length, and plant height, compared to the control and neat urea.	[138]
Poly(starch/acrylic acid) superabsorbent hydrogel (SAH)	Water	Sunflower under drought stress	The shoot and root length increased by 49.84% and 5.35%, respectively, compared to the absence of SAH. Growth parameters and photosynthetic pigment levels in sunflower plants grown under drought conditions were reduced without SAH. However, hydrogel application enhanced photosynthesis.	[139]
Cassava starch-graft-poly(acrylamide) copolymer	Water	Chili plants	The addition of hydrogel to the soil, combined with watering every three days, increased soil porosity and water retention, maintained higher nutrient levels, and preserved the soil’s biological properties. This treatment resulted in better plant growth compared to the control, which received daily watering without the polymer.	[140]
Starch-grafted-poly(sodium acrylate)	Water	Melon (*Cucumis melo* L.) seeds	Field experiments were conducted to evaluate the effects of hydrogel quantity, substrate type (sandy soil and coconut fibers), and soil type (sandy soil and clay soil) on various plant growth parameters. Overall, plants grown in coconut fibers exhibited the highest growth (5.60 cm) compared to those cultivated in sandy soil (4.12 cm).	[141]
Corn starch—urea extrusion material composite	Urea—melamine	Sweet corn	Greenhouse trials revealed that melamine plays a crucial role as a structural modifier, enhancing the effective utilization of N from urea in maize pot experiments. Additionally, it was observed that N-melamine remained unavailable during the first 60 days of the trial, indicating that the lower amount of N released (solely from urea) was more efficiently utilized by plants treated with composite material.	[142]
Starch-g-poly(acrylic acid-co-acrylic amide) (SBS-g-P(AA/AM)) as the skeleton and urea-formaldehyde oligomers	Urea	Maize	N release experiments confirmed that SBS-g-P(AA/AM)-UF provided a gradual N supply in the soil. Compared to conventional urea and UF fertilizers, maize yield increased by 20.3% and 9.7%, respectively, with the application of SBS-g-P(AA/AM)-UF.	[143]

In the previous experiment, the researchers used only 0.05 g of the dose per spot, reducing the use of fertilizer during the experiment. However, the amount of hydrogel was not evaluated in this research, which presents a gap in the literature. This is important because the fertilization dose and the plant’s growth stage are key factors, as they need to align the nutrient requirements of the plant with the nutrient release of the material. For instance, during the germination stage, plants require lower nutrient levels, but in the vegetative stage, the plant’s macronutrient requirements increase. Thus, the SH modified with acrylic acid becomes a strong alternative for nutrient release in short-cycle crops, as it releases approximately 20% of the urea in the first 5 days, supplying nitrogen during the germination phase. Following this, the release of urea from 20% to 100% supports the plant during the vegetative stage [134]. However, this material could be limited for optimal nitrogen supply in long-cycle crops with a vegetative stage longer than 3 months, requiring adjustment in the fertilization process. To determine the optimal dosage, it is important to characterize the environmental conditions, including temperature, moisture, and microbial activity in the soil, as these parameters directly affect nutrient release.

To reduce the urea release time from starch–poly(acrylic acid) hydrogel, and to make it suitable for long-cycle crops, wax and NR have been incorporated to protect the material and inhibit water permeation (BHWCU) [112]. In vitro urea release experiments showed that the BHWCU material released 40% of the nutrient in water within 100 h (approximately 4 days), and the same release percentage was observed in soil environments over 30 days. However, urea release is influenced by factors such as pH and temperature. After 100 h, the material released 30%, 40%, and 70% of urea at pH 4, pH 7, and pH 10, respectively. Additionally, increasing the temperature from 20 °C to 40 °C resulted in an increase in cumulative urea release from 30% to 55% at 100 h. This effect is likely due to the increased thermal flexibility of the polymer chains, which expand as the temperature rises, accelerating the release of urea. In plant growth experiments with chili plants, those treated with the BHWCU formulation exhibited healthier growth, with broader, thicker, and darker green leaves compared to plants treated with conventional urea. Thus, the BHWCU formulation effectively supplies urea to the plant, fulfilling its nutrient requirements.

Note that the experiment was conducted during the vegetative stage for 6 weeks, providing the nutrient requirements for the plants. However, according to the experiments on nutrient release in soils, the release of the nutrient can be modulated by the proportion of NR to polymer. A higher amount of NR (9:1 with the polymer) reduces nutrient release, with only 40% of the urea being released within 30 days. This proportion is suitable for long-cycle crops. On the other hand, a 2:8 ratio of NR to polymer induces a more rapid release, with 80% of the urea being released in the soil, making it an optimal material for short-cycle crops [112]. Thus, the optimal dose can be adjusted according to the plant growth conditions.

**Figure 8:**
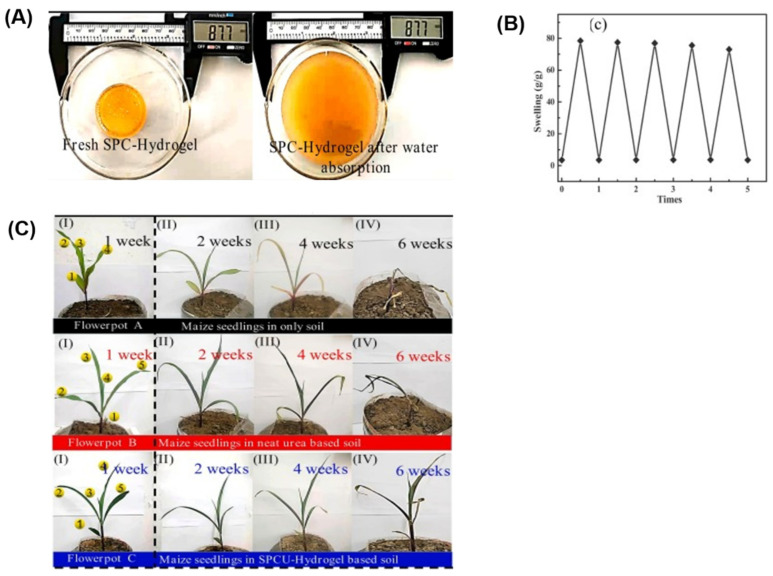
(**A**) Dry and swollen SPC hydrogel. (**B**) SC of SPC hydrogel after six hydration and dehydration cycles. (**C**) Effect of neat urea and SPCU-hydrogel on maize seedling growth. Modified from [136].

To develop materials for controlled fertilizer delivery, other monomers, such as acrylamide, were copolymerized with corn starch to encapsulate urea [136]. Starch phosphate carbamate (SPC) was formed in the presence of urea, NaH_2_PO_4_, and Na_2_HPO_4_, and then copolymerized with acrylamide using (NH_4_)_2_S_2_O_8_ as the initiator, resulting in SPCU-hydrogel. The hydrogel exhibited an increase in volume upon absorbing water (Figure 8A). Its SC showed only a slight decrease after six cycles of water absorption and dehydration (Figure 8B). Column leaching experiments were conducted to determine the urea release profile, which was compared with SH (NSU-hydrogel) and neat urea. In sandy loam soil, pure urea released nearly 100% within 24 h. In contrast, the hydrogels exhibited slower release, with NSU-hydrogel releasing 100% of the urea in 25 days, and SPCU-hydrogel in 35 days. This difference is attributed to the inherent properties of esterified SPC, such as high viscosity and hygroscopicity. The ability of SPCU-hydrogel to support maize seedling growth in soil was tested over six weeks (Figure 8C). The SPCU-hydrogel-treated seedlings thrived for at least five weeks, while the control pots (with or without urea) only lasted two weeks without water addition. The SPCU-hydrogel proved to be more favorable for plant growth, potentially contributing to improved crop productivity.

SH composites incorporating nanozeolites (NZs) have been developed to enhance nutrient absorption and regulate nutrient release rates [144]. Hydrogel beads were synthesized using gelatinized sago starch, CS, and NZ, with TPP as a crosslinking agent (SCHNZ-TPP hydrogel). The material was tested for P and urea release behavior in deionized water and in *Philodendron* sp. plants [138]. After 14 days, the hydrogel composite released 64% of P and 41% of urea in water. This behavior is attributed to interactions between NH_3_^+^ in urea and Al in NZ, whereas P (PO_4_^2−^) does not interact with NZ, leading to a higher release rate of P compared to urea. The hydrogel containing urea demonstrated a significant effect on *Philodendron* sp. growth, enhancing the growth index and chlorophyll content compared to the control. This confirms its potential as an effective slow-release fertilizer. Other nanomaterials, such as NCNPs incorporated into starch-g-poly(styrene-co-butyl acrylate), have also been explored to optimize urea release from SHs. Salimi et al. (2021) evaluated these materials in greenhouse tomato production, testing different NCNP concentrations (0–1% *w*/*w*) [137]. Their findings indicate that NCNPs play a crucial role in improving tomato plant growth while reducing the N release rate. The formulation containing 1% NCNPs resulted in 13.75% greater greenery in tomato plants compared to conventional urea and other tested formulations. This effect was further reflected in an increased number of leaves and higher chlorophyll content.

## 5. Conclusions, Challenges, and Outlook

SHs are classified as non-toxic and biocompatible materials, with the ability to absorb and retain water, making them attractive for water supply in arid soils. Additionally, SHs can encapsulate fertilizers to enable a slow release in crops. They can be synthesized through graft copolymerization, as well as chemical and physical crosslinking. However, SHs based on the polymerization of acrylic monomers have shown significant scientific advancements, leading to the development of methodologies that utilize rotating drums and extruder machines for large-scale production. According to in vitro assays (nutrient diffusion from the polymeric matrix into the solution), SHs exhibit a high release rate. Nevertheless, in agricultural applications involving soils and plants, the release rate decreases, extending up to 30–60 days. This means SH-based fertilizers are a viable method for promoting seed and plant growth. Despite the advantages of using starch for controlled nutrient release, there are still some drawbacks and challenges to overcome in this field:

1. Most studies on SHs have focused on the release of N sources, with limited research on P, K, and micronutrients. In particular, researchers need to focus on developing versatile SHs capable of promoting the simultaneous release of NPK and water to plants.
2. Many researchers have concentrated on SH production at the laboratory scale, with few studies exploring large-scale applications in crop production. As a result, there is limited information on application costs and feasibility, making it difficult to compare SH-based fertilization with conventional methods.
3. The limited information on the application costs of SHs in soils also creates a knowledge gap in areas such as storage, mass production, and standardized protocols. Furthermore, the high biodegradability of SHs presents a challenge; research is needed to develop slow-release nutrient systems with high nutrient loading to reduce application frequency in soil. Additionally, new formulations should aim to improve the resistance of SHs to microbial degradation, which can otherwise lead to a rapid release of nutrients.
4. Although SHs enhance the biodegradability of hydrogels formed with synthetic polymers, they exhibit low water retention under salt stress conditions, which negatively impacts continuous nutrient release in soil applications. Therefore, further research is needed to develop new SHs with improved performance under saline conditions. These materials must also be cost-effective to ensure adoption by farmers. For instance, the use of agricultural residues is emerging as a promising strategy to produce affordable SHs without requiring complex production processes that could increase the final product cost.

## Abbreviations

The following abbreviations are used in this manuscript:

AP	amylopectin
AM	amylose
KPS	ammonium persulfate
CM	carboxymethyl
CS	chitosan
CA	citric acid
Dg	degree of grafting
DS	degree of substitution
DMSO	dimethyl sulfoxide
EH	epichlorohydrin
GA	glutaraldehyde
GYX	glyoxal
MA	malic acid
MBA	methylene bisacrylamide
M_w_	molecular weight
N	nitrogen
NCNPs	natural charcoal nanoparticles
Nps	nanoparticles
NR	natural rubber latex
NZs	nanozeolites
P	phosphorus
PEC	polyelectrolyte complex
PVA	polyvinyl acetate
K	potassium
REX	reactive extrusion
SAH	starch-modified poly(acrylic acid)
SCM	starch–carboxymethyl
STMP	sodium trimetaphosphate
SPC	starch phosphate carbamate
SHs	starch-based hydrogels
SC	swelling capacity
SW	swelling ratio
TPP	tripolyphosphate
